# The Effects of Enrichment on Zoo-Housed Scarlet Ibis Behavior

**DOI:** 10.3390/ani14131903

**Published:** 2024-06-27

**Authors:** Patrícia Rachinas-Lopes, Inês C. Rocha, Tiago Dias, Maria Tavares, Ricardo Neto, Carla Flanagan, João Neves

**Affiliations:** 1 MARE—Marine and Environmental Sciences Centre/ARTNET—Aquatic Research Network, Faculdade de Ciências, Universidade de Lisboa, 1749-016 Lisboa, Portugal; 2 Ispa—Instituto Universitário de Ciências Psicológicas, Sociais e da Vida, Rua Jardim do Tabaco, 34, 1149-041 Lisboa, Portugal; 3 Zoomarine Algarve—Portugal, EN 125 Km 65, Guia, 8201-864 Albufeira, Portugal

**Keywords:** environmental enrichment, behavior, scarlet ibis, *Eudocimus ruber*, captivity, Zoomarine Algarve

## Abstract

**Simple Summary:**

Environmental enrichment is a dynamic process used by zoological institutions to provide stimuli that respond to the needs of the animals and increase their quality of life. This research explores how environmental enrichment affects the behavior of a group of scarlet ibises in a zoological context. Despite being a popular species under human care, the scarlet ibis has not been the subject of many studies. The results show that some behaviors changed when the enrichment devices/strategies were presented. The use of brushes and changes in the water volume of the lagoons showed higher activity and less stationary behaviors compared with the other strategies. This study highlights the importance of adjusting the strategies so the stimuli can be not only biologically significant but also efficient in the long term.

**Abstract:**

Good zoo animal welfare is commonly promoted with environmental enrichment; however, some species are less likely to be offered enrichment than others. This study tested the effect of enrichment on a group of scarlet ibises from Zoomarine Algarve, Portugal. The study consisted of a first baseline condition, followed by four types of enrichment displays with individual presentations, a post-enrichment condition, and a post-enrichment with all enrichment types presented simultaneously. The enrichment types chosen were physical, with a tidal simulation in the lakes of the enclosure; nutritional, presenting mussels in a plastic mesh tube; sensory, by playing scarlet ibises calls; and tactile, with brushes through the habitat. The data collection was performed for 21 days between January and March 2021, 3 times a day, using scan sampling and instantaneous time sampling every 2 min. The comparison between conditions revealed that most behaviors showed similarity between the baseline and post-enrichment conditions, suggesting that after removing the enrichment, the behaviors returned to their initial baseline. It was also found that each enrichment influenced different types of behaviors and these behaviors also changed depending on the time of day. Exploratory behavior was only associated with the presence of enrichment, and vocalizations were only heard after the sensory enrichment was performed. This study demonstrates that the use of physical and tactile enrichments increased activity and decreased stationary behaviors in this group of ibises and may be used to improve their lives in zoological contexts if included in the housing and husbandry protocols.

## 1. Introduction

Modern zoos and aquariums (hereby zoos) have been a constant over the years. Their focus, however, has shifted from mere entertainment and visitor satisfaction to a stronger commitment to education and conservation, with a continuous increase in concern for animal welfare [1,2]. 

Animal welfare has been defined in different ways throughout the years, based either on health (biological function), natural way of living, or animals’ feelings [3]. One of the most widely used definitions of welfare refers to the state of the individual while attempting to cope with the environment [4]. An animal is influenced by inputs (resources available, husbandry, space, etc.) and outputs (observed through behavior), which resulted in the creation of the Five Domains model, which represents the areas of nutrition, environment, health, behavior, and affective experience domains—mental state—with positive and negative states associated with each area [5,6,7]. The ultimate goal for any animal under human care is to have positive states/experiences exceeding the negative ones [8], and evidence-based approaches to animal husbandry provide the appropriate inputs to result in increased positive states and outputs [7]. One key area of captive animal husbandry where modern zoos have been working to increase positive states based on scientific evidence is through housing and husbandry practices, namely the use of environmental enrichment techniques [5,9,10]. 

One of the accepted definitions of environmental enrichment (EE) is the improvement of the biological functioning of animals under human care that results from modifications to their habitat [11]. According to Bloomsmith and colleagues, EE can be divided into five types of enrichment: social, cognitive, structural, sensory, and nutritional, each of which encompasses different categories [12]. The EE should be species-specific and created with knowledge of the ecology and behavioral needs, where the effects are evaluated, providing stimuli that enhance the opportunities for the animals to experience positive welfare [13,14,15,16]. However, because these practices have been widely implemented in zoos, it is expected that there is always a benign effect, and their impact on the welfare of the animals is not always questioned [9]. Some of the strategies are still lacking the lasting effects evaluation that may provide long-term welfare improvements to the animals [15]. One of the problems with this approach is the anthropocentric view of which species are in greater need and which conditions are considered essential for the species to thrive in a zoo context [9]. The most obvious example of this is that most of the studies involving EE are applied to terrestrial mammals, especially non-human primates and carnivores, even though birds tend to be present in larger numbers in zoological institutions [9,10,17]. Birds are very resourceful animals [18], and as a result, they are subject to boredom due to monotonous and predictable routines [17,19]. A species in captivity has important factors of its development removed and/or substituted. The consequences include a reduction in choice that can modify behavior patterns and decrease the psychological welfare of the animals [20], extinguishing some behaviors and/or increasing inactivity [19]. The most common enrichment used by birds tends to be nutritional enrichment [21]. Studies showed that food-based enrichment proved to be effective for bird species, reducing the expression of abnormal behavior like eating feces and pacing, increasing walking, flying, and foraging behaviors, and decreasing problems like bumblefoot [22,23]. Birds’ inactivity can be related to poor foot health, and increased activity and movement across different substrates may help reduce foot lesions [24].

The scarlet ibis, *Eudocimus ruber*, least concerned (LC) species according to the IUCN Red List [25], is a colonial waterbird that can be found in the northern part of South America, mostly in mangrove swamps and coastal lagoons. These birds live in colonies of up to 60 individuals, with an average of 15 to 20, frequently mixed with other species, namely, herons and spoonbills. They usually leave the roost in the morning to go feed on the mud flats, often probing and pecking in the water to catch their prey and fly back in flocks before sunset [26,27]. This is a defensive species that will aggressively fight for space as a consequence of having prey stolen by other birds, even though they do the same for other species [28]. Breeding season usually initiates in mid-spring, with males performing courtship displays and building nests in trees. This behavior culminates with the increased water levels in their habitat, favoring the feeding and growth of the offspring [29]. In the wild, ibises feed mainly on small crabs and mollusks but also on aquatic insects, small fishes, snails, and green algae [26]. Their long bill allows them to forage in shallow and muddy waters [29], where they feed primarily by non-visual probing, walking slowly, and pecking prey [28].

Although this species is common in captive environments, it has been present in very few studies in this context. Antas [30] focused on its breeding behavior, as did Spil and colleagues [31], who also made a small reference to their daily activity, verifying that was similar to the animals in the wild, but none of them focused on the general behavior of this species. Thus, this study appears to be one of the few attempts to investigate the behavior of this species in a zoo, with the main goal of evaluating the effects of enrichment on the behavior of a group of scarlet ibis. Knowing which types of enrichments contribute to the increase in the behavior repertoire and activity patterns in a specific group of ibises may help not only better understand their behavior in a zoological context but also provide more tools and information on how to improve their welfare in a zoo by updating husbandry protocols and/or improving the habitat design.

## 2. Materials and Methods

This study was reviewed and approved by Zoomarine’s Science Committee (Project nº ZM_2020ID07). This project followed careful procedures to minimize disturbance to the animals. Since the animals were not managed by the researchers and had minimal changes in their routines, any other ethical approval was waived.

### 2.1. Study Population and Location

**Figure 1:**
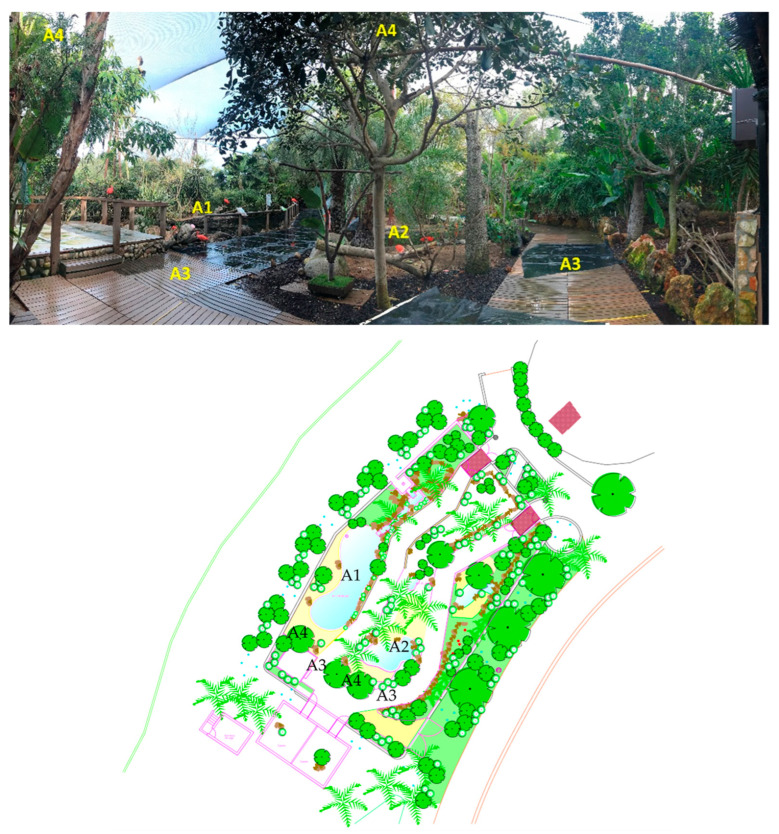
Americas habitat with the respective areas used by the group of ibises (**above**). A1 (Area 1)—large lake area (exit side); A2 (Area 2)—medium lake area (central zone); A3 (Area 3)—walkway accessible for the public to walk; A4 (Area 4)—upper part of the trees. Map of the complete habitat (**below**).

This study was developed at Zoomarine Algarve, a zoological and theme park in Guia, Algarve (Portugal), from November 2020 to March 2021. Zoomarine has a wide variety of species of birds that were distributed in three areas: “Wings of the World” (birds of prey), “Americas” (a mixed species habitat), and “Magic Rainbow” (with tropical bird species). The data collection was conducted in the “Americas”, a 730 m^2^ immersion habitat where visitors can walk through and find species from the American continent (Figure 1). This habitat recreates the natural environment of these species through the presence of four lakes of different sizes, a waterfall, and vegetation. 

A group of 16 scarlet ibis, composed of 11 adults, 4 offspring from 2020, and 1 juvenile from 2019, share this habitat with eleven other bird species and two reptile species (see Table S1 for more details). Since the group of ibises remained in the areas described in Figure 1, their behavior was always trackable by the experimenter, even when they were in the trees (areas A4).

In terms of hierarchy rank in this habitat, in general terms, the most dominant groups seem to be the giant wood rail (*Aramides ypecaha*) and the guira cuckoo (*Guira guira*). However, in terms of food competition, ibises seem to be dominant, as they are described by literature as defensive to avoid food stolen by other bird species [28]. 

In this zoological context, ibises eat a total of 2 kg per day of a mixture of meat and fish, separated into 2 daily feeding sessions (morning and afternoon), which normally include quail or chick, capelin, smelt, herring, and mackerel. They are also fed 300 gr daily of a mixture of granulate for ducks and flamingos from the Versele-Laga brand in dishes distributed in different areas of the habitat. The other species in the habitat would have access to 1 kg of the same meat and fish mixture, also divided into two feeding sessions.

### 2.2. Behavioral Data Collection and Enrichment Schedule

The study started with two weeks of preliminary observations with the ad libitum sampling method [32], in November 2020, to study the dynamics of the group and become familiar with the behavior of the ibises. Since there was no known ethogram for the Scarlet ibis, the ethogram published by Spiezio and colleagues [33] was adapted to the current species. These preliminary observations had two main goals: (1) to register how many different behaviors were performed by the group of ibises, and (2) as an adaptation period from the birds to the experimenter. All behaviors registered were then compared with the ethogram, and a detailed description of the new observed behaviors was written. These new behaviors were then added to the ethogram used during the data collection (Table 1).

The data collection was conducted between January and March 2021. As it was not possible to distinguish individuals from each other, the scan sampling method and instantaneous sampling were used [32], every 2 min, three times a day with a one-hour duration: morning, from 10–11 am; midday, 1:30–2:30 pm; and afternoon, 4–5 pm. The data collection was divided into four conditions: condition 1—baseline; condition 2—enrichment; condition 3—post-enrichment; and condition 4—post-enrichment with devices. The experimental design consisted of an ABAC design, where A represents the behavioral data without devices; B is the enrichment condition [34], with the different types of enrichment; and C is the presentation of all types of enrichment together (Table 2). 

#### 2.2.1. Baseline

The baseline condition was carried out to observe and quantify the behavior of the group of individuals throughout the sessions and to observe how they performed their daily routines. This condition lasted 3 consecutive days, from 6th to 8th January with a total of 9 h of observation.

#### 2.2.2. Environmental Enrichment

Since information on EE for this species is rare or even unknown, four types of enrichment—physical, nutritional, sensory, and tactile—were selected. This selection was made to include different stimuli to understand which one could be more relevant to this group of birds. 

Due to the pandemic constrictions and security measures inside the marine park at that moment, this condition was performed with an interval of 5 days between enrichment types, with 3 non-consecutive days of observation for each enrichment type, totaling 9 h of observation for each type (3 h in the morning, 3 midday, and 3 in the afternoon). 

**Figure 2:**
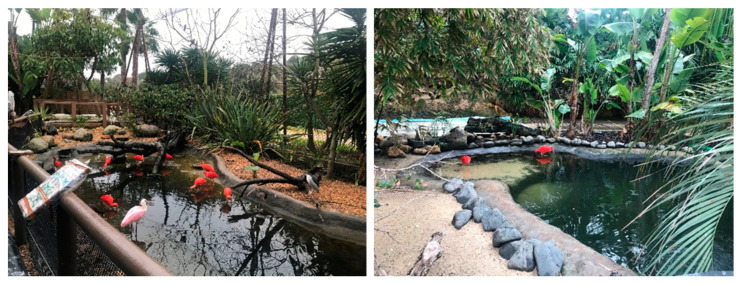
Tidal simulation in the two larger lakes, from left to right, in area 1 (low tide) and area 2 (intermediate tide), respectively.

**Physical enrichment**, from 26th to 30th January, consisted of tidal simulation in the two larger lakes with the main goal of promoting the exploration of the lake and feeding behaviors. EE was chosen as these animals are aquatic and migratory species that gather in flocks during high tide in their natural habitats [27]. It was stipulated that during the morning, the lake should simulate the low tide and, in the afternoon, the high tide. Thus, in the morning, 15 min before the beginning of the observations (9:45 a.m.), the animal care staff enabled the system to decrease the amount of water in the lake (taking 1 h to descend), reaching the lowest point at 10:45 a.m. and so remaining until the end of the morning’s data collection. As the lake was not very deep, to simulate a rising tide, it was decided that in the middle of the day, the depth of the lake would be intermediate, and only in the afternoon, the high tide would be reached, to simulate the 6 h difference between low and high tide. Thus, at the beginning of the afternoon’s observation, the system was turned on so the level of water in the lake could slowly increase. The lake took approximately half an hour to reach its maximum level. The simulation of these tides was only carried out on the days of the observations (see Figure 2 for an example and Figure S1 for a complete sequence of high, intermediate, and low tides, for both lakes).

**Figure 3:**
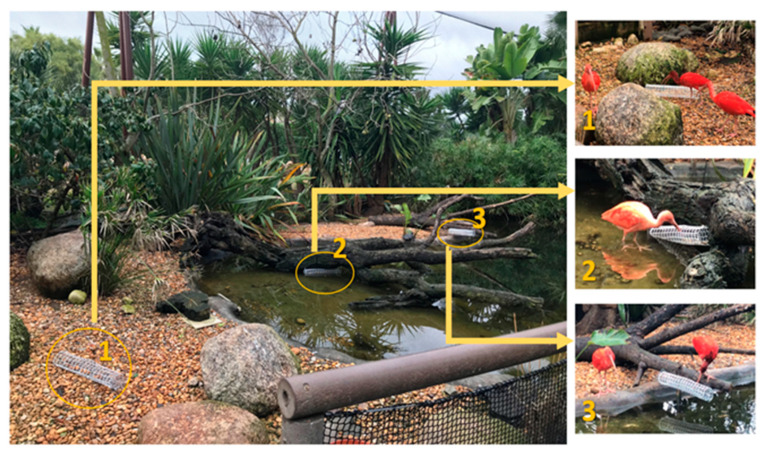
Example of the nutritional enrichment distributed in the habitat (1—ground; 2—half-submerged in the water; 3—above water).

For **nutritional enrichment**, from 5 to 9 February, plastic mesh tubes with mussels were placed by the caretakers in the habitat. This EE was chosen as mussels are not included in these birds’ diet, but they do normally eat them in the wild (see [26]). The mussels are a novel food item for these individuals, and this enrichment also allowed the food to be presented differently to increase foraging and exploratory behaviors. During the days of nutritional EE, the device with mussels substituted the morning feeding, to avoid an increase in the daily calories of the birds’ diet. A total of six devices were used, including one half-submerged in the water (Figure 3). The devices were provided at the beginning of each of the three daily sessions and removed after they ended.

For **sensory enrichment**, from 15 to 19 February, scarlet ibis calls downloaded from the xeno-canto.org website were introduced. This EE was chosen as Ibises are a very social species and feel safe and comfortable in large flocks [27]. The study conducted by Clark et al. (2012) found an increase in the breeding behaviors of the northern bald ibis (*Geronticus eremita*) after some breeding calls playback [35]. Although the group size of the present study falls within the average number of individuals, this enrichment had the intention of studying if the ibises would vocalize more if they could hear other individuals from the same species, simulating a bigger flock.

A 10 s call was chosen and then transformed into a 1 h loop with 5 random pauses. This sound was only presented during the data collection, so the reaction of the animals was quantified. For this purpose, a sound emitter was placed in a central area of the habitat.

**Figure 4:**
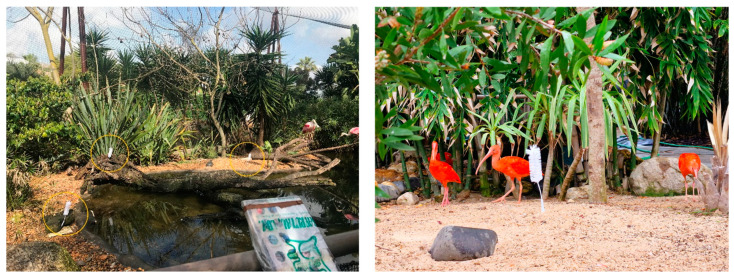
Tactile enrichment, in circles on the left, throughout the habitat.

**Tactile enrichment**, from 25 February to 1 March, consisted of 6 brushes distributed through the habitat, on the ground and trunks (Figure 4). This enrichment, although not a natural stimulus for the animals, was chosen to study how the animals react to novel and unknown stimuli in their habitat. It was also included to stimulate preening by rubbing their bodies and heads against the brushes. These brushes were only placed during the observation periods.

#### 2.2.3. Post Enrichment

This condition lasted for 3 consecutive days, from 9 to 11 March, with a total of 9 h, and the observing conditions were similar to the baseline.

#### 2.2.4. Post Enrichment with Devices

In this condition, from 19 to 21 March, all EE types were applied simultaneously, and, contrary to the enrichment condition, the tactile enrichment devices remained in the habitat throughout the entire day. The physical enrichment (tide simulation) remained the same as used in the enrichment condition, with low tide in the mornings and high tide in the afternoons. Due to calorie intake and battery charging, the nutritional and sensory enrichments also followed the same plan as in the enrichment condition. At the end of the day, all devices were retrieved from the habitat to sanitize and were placed in position the following day. This condition lasted for 3 consecutive days, for a total of 9 h.

### 2.3. Data Analysis

To avoid giving different significance weight to behaviors that had a very low number of occurrences, behaviors that presented less than 10% in number compared to the most observed behavior were discarded from the analyses.

To perform the behavior comparisons among conditions, a Friedman’s test was performed at a significance level of *p* < 0.05, with the Wilcoxon test to study the pairwise comparison. Kendall’s W was used to report the effect size of the behavior comparisons. To study the influence of the “Time of Day” (morning, midday, and afternoon), a Kruskal–Wallis H test with Bonferroni correction was performed.

All the statistical analyses were performed using the IBM SPSS Statistics for Windows, Version 29 software (IBM Corp; Armonk, NY; USA).

## 3. Results

The behaviors observed for the group of ibises were consistent with the ones described in the ethogram by Spiezio and colleagues [33]. However, five more behaviors were observed. Table 1 includes the behaviors observed for this species, including the new behaviors’ descriptions.

Because of the description of some behaviors, the name was changed for the ibises ethogram to better reflect what was observed. This includes the alertness and attentive behaviors, in the Spiezio and colleagues [33] ethogram, which were transformed into one for this group and named attentive behavior. The same happened to “Comfort behavior”, which was changed to preening; and “Manipulation/play”, which changed to foraging.

Although new behaviors were observed and described, only behaviors that had more than 10% of occurrences compared with the most observed behavior were included in the data analysis. This means that all behaviors with less than 116 occurrences were discarded (the most observed behavior was preening with 11,555 occurrences). The social agonistic behaviors were observed across all conditions, however, at a low number, and so the three behaviors from this category were collapsed into one general category, named General Agonistic Display. The behaviors included in the analysis were: attentive, preening, flight, walking, maintenance, foraging, resting, exploration, stationary behavior, and general agonistic display.

Exploration was only observed in enrichment conditions, in which the bird uses its bill to explore new parts of the lake that did not have access to it before, pulling algae from the bottom of the lake (physical enrichment at low tide) or exploring new items in the habitat (nutritional and tactile enrichment).

Interaction between the group of ibises and any other species in the habitat was quantified; however, it was not included in the analysis as only 81 occurrences were observed, including social and agonistic displays.

Since it was not possible to quantify the vocalizations in number, this behavior was analyzed as presence or absence. This behavior was only observed after the sensory EE type.

No abnormal or stereotypical behaviors were reported during this data collection, and so they were not included in this ethogram version.

### Comparisons among Conditions and Different Types of Enrichment

The behaviors that significantly differed between conditions on the Friedman’s test are presented in Table 3.

Attentive behavior showed a decrease in the physical EE type, with an increase for the Nutritional and sensory types, going back to baseline values in both post-enrichment conditions. Flight decreased during the sensory EE (Table 3). These variations are not explained by the time of day, as this variable did not show significant differences for both behaviors (F (2, 7812) = 5.353, *p*-value = 0.069 for attentive and F (2, 7812) = 5.805, *p*-value = 0.055 for flight).

Preening had an increase in the physical, sensory, and tactile EE types, and in both post-enrichment conditions. On the other hand, exploration showed increased activity in the physical, nutritional, and tactile EE types, with the highest values in the last condition, post-enrichment with devices (Table 3). Both behaviors showed significant differences in the time of day (F (2, 7812) = 82.812, *p*-value < 0.001 for preening and F (2, 7812) = 172.401, *p* < 0.001) for all three pair combinations (morning, midday, and afternoon), with an increase in preening in the midday period, and a decrease in exploration towards the end of the day.

Walking showed a decrease in activity when the physical, nutritional, and sensory enrichment types were present, and it was back to baseline values in the tactile enrichment. This behavior showed the lowest values in the post-enrichment with devices. Maintenance behavior showed lower numbers in physical enrichment type and post-enrichment condition (Table 3). It was observed for both behaviors’ significant differences in the time of day (F (2, 7812) = 173.341, *p*-value < 0.001 for walking and F (2, 7812) = 241.152, *p*-value < 0.001 for maintenance), between the pair morning–afternoon (*p*-value < 0.001) and midday–afternoon (*p*-value < 0.001), with a decrease in activity towards the end of the day.

Foraging showed a decrease in activity in nutritional, sensory, and tactile EE types and both post-enrichment conditions (Table 3). This behavior showed significant differences in the time of day (F (2, 7812) = 633.808, *p*-value < 0.001) for the pairs morning–afternoon and midday–afternoon, with a decrease in activity towards the end of the day (*p* < 0.05).

Resting showed an increased number towards the progression of the data collection, with a bigger increase in tactile enrichment (Table 3). This behavior showed significant differences in the time of day (F (2, 7812) = 407.548, *p* < 0.001) for the three pair combinations (morning, midday, and afternoon), with an increase towards the end of the day.

Stationary showed a decrease in numbers in physical, sensory, and tactile EE, as well as in post-enrichment with devices (Table 3). This behavior seems to be affected by the time of day (F (2, 7812) = 268.430, *p*-value < 0.001) for the pairs Morning-Afternoon and Midday-Afternoon, with an increase towards the end of the day (*p* < 0.001).

Vocalizations were heard only after the presentation of the sensory EE, having a small increase in both post-enrichment conditions (Table 3) with differences in the time of day (F (2, 7812) = 13.102, *p*-value = 0.001), namely in the midday–afternoon pair (*p*-value = 0.003), with an increase in vocalizations towards the end of the day.

General agonistic display showed zero interactions during tactile Enrichment with significant differences in the time of day (F (2, 7812) = 8.248, *p* < 0.016), namely in the pair morning–midday (*p* < 0.001), with a decrease in activity (*p* = 0.027).

## 4. Discussion

To analyze the effects of environmental enrichment on the behavior of a group of scarlet ibises under human care, a study was conducted at Zoomarine Algarve. The present study analyzed the behavior of a group of 16 birds using four types of EE: physical, nutritional, sensory, and tactile. To understand the behaviors exhibited by this species, an ethogram of the northern bald ibis [33] was used and adapted. The results showed that physical and tactile EE were the ones increasing the activity of the birds and reducing their stationary behaviors. Exploration was observed only in the presence of EE, and vocalizations were heard only after the sensory EE. No abnormal behaviors or stereotypies were observed during the data collection.

### 4.1. Comparisons between Conditions and Types of Enrichment

The EE techniques applied in this study showed an effect on some behaviors exhibited by a group of scarlet ibises, such as attentive behavior, preening, walking, maintenance, foraging, resting, exploration, flight, general agonistic display, stationery, and acoustical behaviors such as vocalizations.

Comparing the conditions and types of enrichment, it would be expected that behaviors in baseline and post-enrichment conditions would have a similar response since the latter is often considered a second baseline [34,36]. However, this was not always the case, verifying that preening, maintenance, resting, foraging, and vocalizations showed significant differences between the two conditions. Maintenance and foraging were the only behaviors showing a decrease between baseline and post-enrichment conditions. The increased photoperiod during data collection (January to March), although not very accentuated, may have influenced some behaviors, since, in natural environments, ibises tend to be more active in periods of lesser light during the day, early in the morning and before nightfall due to foraging and feeding activities [26]. In nature, ibises typically feed before 7 a.m. and after 5 p.m. [31], a condition that in zoos, most of the time is not possible to apply as the schedules and routines are dependent on the caretakers, and usually occur in the 6–8 h of daylight, which may also not be biologically significant for some species [37]. This may explain the fact that, in the present study, active behaviors like walking, maintenance, and exploration showed a decrease in occurrences towards the last session, but with a possible increase later in the day, closer to sunset. Preening was unexpectedly found more in the midday session, which contradicts what is known about this behavior for this species [33,38]. Since under human care, the routines are normally earlier than in nature, e.g., feeding time, the increase in light during the day can make the birds more likely to preen themselves, taking advantage of the light to keep themselves warm, rather than eating from the feeder. It could be interesting to extend these observations to the period in which animal care staff are absent to study how the behavior may vary.

The lack of significant differences observed in the behaviors between baseline and post-enrichment conditions may be due to a predictable environment, meaning that these animals do not need to be alert or searching for new stimuli, individuals, or predators, as opposed to when something new is added to the environment (EE types) that stimulates their curiosity, as also observed by other studies [34,36,39]. Exploration is a behavior of curiosity, elicited in the presence of new stimuli [39,40], and hence it is no surprise that the animals display it only in the presence of EE. This may also reveal that after the removal of the stimuli, the animals tend to return to their previous patterns, exhibiting more resting behaviors [34,36], a result also found in the present study.

The physical enrichment (i.e., tidal simulation) increased activity levels by increasing the occurrences of preening and exploration behaviors, decreasing the stationary behavior. Preening is described in other studies as a comfort behavior as a strategy to refrain from ectoparasites or decrease local irritation [33]. The increase in this behavior for this EE, the highest occurrence for this behavior in the data collection, may be related to the fact that this EE made accessible a part of the lake that was interdicted before, and so birds explored the waters, having the necessity to preen themselves after to avoid any irritation and/or ectoparasites. The increase in these behaviors is concordant with the findings of other studies [33]. Even though, biologically, the behaviors that were expected to increase in this scenario are foraging, probing, walking, and wading [41], as far as we know, animals never saw the lakes with less water, which may greatly increase the exploration of the “new” habitat rather than walking, foraging, or eating. Taking into consideration the ecology and biology of this species, this enrichment is important as it can function as an environmental cue for breeding season and may enhance more social interactions since, in the natural environment, these colonial birds are social species, especially during nesting and feeding periods in shallow and muddy waters [26,28,29]. Another explanation for the decrease in maintenance behavior is the fact that “Americas” is a naturalistic enclosure with rocks, trees, grass, and other natural characteristics that may be enriching for these ibises, stimulating their feeding behavior naturally [26].

The increase in attentive and exploration behaviors and decrease in foraging, for the nutritional EE may be explained by the novelty of the device presenting the food type, which is also a novelty for these birds. Since this EE was in the form of a mesh tube with mussels, it called for greater interest and attention, thus also leading to immediate consumption after its presentation. This fast consumption may explain the apparent decrease in foraging behavior, leaving the birds without mussels close to the beginning of the session and an empty mesh tube to explore during the remaining session time. In a study with orangutans, when presented with a novel food, the most curious animals quickly engaged with it [42]. This decrease may also be because food is easily available to them through feeders and scheduled feeding sessions, as already noted by other studies in numerous species (e.g., [43,44,45,46,47]). In the wild, scarlet ibises feed mostly on small crabs and molluscs [26], which is not normally included in the diet of this species in this zoological context. The change in behavior and the rapid consumption of the food in this case suggest that the insertion of a food type, that they have access to in the wild, may be an important addition to husbandry protocols and diet as it represents a more naturalistic and ecological approach for this species.

The biggest increase in attentive behavior was observed during the sensory EE type. Normally, providing birds with vocalizations aims to elicit vocalizations and searching behaviors [41]. In this study, it was observed that the sounds caught the attention of the ibises and may be causing a feeling of a bigger group, since these calls may, for example, function to maintain group cohesion (e.g., through calling to foraging sites) [48], and hence they were more attentive and looking for the presence of more birds. Scarlet ibises gather in large flocks in the wild, and these high numbers serve not only for sharing feeding areas [27,28], but also as a protective measure against predators, as they function as camouflage [28]. In this zoological context, even in a controlled environment, the use of playback calls may give the sense of safety that they naturally find in a higher number of individuals. On the other hand, the increased attentive behavior levels may also be indicative of stress [49] regarding this new sound in the habitat that may, or may not, contain biological information for these birds. Being attentive is a consuming activity for these birds, and it would represent less time in other activities, such as foraging and social/reproductive behaviors [50]. More studies are needed to make sure this attentive behavior is due just to the novelty of the sound and not to increased stress, which may decrease with time. In the present study, it was also observed that ibises started to vocalize after this EE, which may suggest some interest or curiosity from the birds. However, more studies are needed to understand whether this increase in vocalizations was a consequence of the EE or a result of seasonal change, as the breeding season starts in mid-spring [26,51].

In the case of tactile EE, as it is something completely different from what these birds were used to, there was a greater tendency to explore what was new [40]. However, it was also observed that, in the presence of this EE, the resting behavior had its highest values, which may indicate that, despite being something new, it may eventually cause habituation (i.e., “response decrement as a result of repeated stimulation” [52,53,54,55]), and the “novelty effect” quickly disappears. For example, the change of individual boxes to social boxes in stallions showed an increase in social interactions but this effect decreased over only four days of experiments, suggesting a habituation to the test conditions [56]. Another study also found that preening and resting behaviors are normally associated [38]. Although with unknown ecological relevance because of their absence in the wild, the brushes were chosen to increase preening behavior, as it was described in other non-bird species that their usage improved grooming and reduced stress (the majority use of this EE is in cattle and cows, e.g., [57]; but there are also studies in turtles, e.g., [58]; and goats, e.g., [59]).

The main goal of the last condition, post-enrichment with devices, was to understand how the group of ibises reacted to all stimuli at the same time. Furthermore, it allows us to see if there is a good reaction to the presence of various stimuli since caretakers intend to continue to apply them in the habitat, sometimes overlapping EE types. Thus, it was found that in this condition, preening, exploration, and vocalizations showed higher occurrences compared with baseline, which resulted in more active animals. However, it should be noted that although the provision of several stimuli simultaneously can produce strong results, it does not allow the distinction of the effect of each one [60] and should be used with caution as it may lead, in the long term, to hyperactivity and/or stress [61]. The use of EE must be continuously reevaluated to ensure it remains biologically relevant for the species and promotes positive welfare states, as suggested by Brereton and Rose (2022) [10].

The low levels of agonistic display observed during this study follow other published studies with northern bald ibis in the wild, where aggression towards conspecifics is not frequent but rather discouraged by touching the opponent with the bill or by displaying threats [38,62].

In the present study, abnormal behaviors were not observed, and together with the fact that ibises were not out of sight during the data collection, this suggests that these birds do not have their welfare compromised as, even though they have places where they can hide from visitors, they normally do not [33].

Brereton and Rose (2019) reviewed the published literature and compared the activity budget of wild and captive flamingos, showing that inactivity is much higher in the latter environment compared with feeding, which is bigger in the wild. Their study suggests that these changes may be related to these birds reaching their daily energetic requirements faster in captivity, a direct result of the permanent availability of food provided on specific schedules [47]. The generally increased activity observed in all EE types with the increase in exploration and the decrease in stationary behaviors is not only beneficial for the birds as a stimulus to avoid boredom [17,19], but it is also important to avoid health problems, such as foot lesions. Inactivity and hard substrates are associated with poor foot health [24], so these results may be useful for the zoos with ibises in their care, regarding which EE may work better to increase activity levels.

The pause of 5 days between each condition and EE type, in addition to being mandatory due to pandemic conditions, was important to counteract the cumulative effects of enrichment. Continuous presentation of stimuli may result in overstimulated birds due to previously being subjected to other EE, which can lead to hyperactivity, stress, or even less exploration of new stimuli, as has already been observed in mice [61]. Thus, this pause allowed the birds to return to their normal behavior before the next stimulus presentation.

### 4.2. Limitations of the Study

The short duration of the study may influence the conclusions obtained, so as a future direction, longer duration studies are suggested to better understand the cumulative effect of enrichment on behavior. Thus, it is possible to see if the observed effects result only from a “novelty effect” and then disappear (which appeared to be the case with tactile enrichment) or if they remain. EE has the function of promoting new behaviors by using new stimuli, which must be ecologically and biologically significant, appealing, and stimulating according to the needs of the species [14]. Therefore, a systematic rotation and re-evaluation of enrichment plans are also essential, either by the way or frequency in which they are provided, or even by providing other enrichment types, to avoid possible habituation.

Nevertheless, it should be noted that just with behavioral analysis, it is not possible to effectively assess whether there was an improvement in welfare related to enrichment. Here only the behavior of the animals was evaluated, verifying that behavioral changes occurred in the presence of enrichment, which is not necessarily an improvement in welfare. The analysis of physiological indicators can provide more data on welfare, and by combining the behavioral data, it can result in a better welfare assessment [63]. Furthermore, animal welfare refers to the state of an individual, being measured at the individual level [4,64], not the group level. In this study, behavioral effects were assessed at the group level as identification of the animals was not possible, and so it is another factor that does not allow us to conclude whether there was an effect on the welfare of the individuals. Future work that intends to evaluate the effect of EE on these birds should focus on individuals.

## 5. Conclusions

In the present study, physical EE seemed to cause important behavioral changes, including an increase in exploration and a decrease in attentive behaviors in this group of scarlet ibises, which implies an increase in activity levels by these birds, beneficial to their physical and mental health. Even though nutritional EE did not show an increase in foraging behaviors, maybe because of the fast consumption of the mussels, it is still worth further investigation. This fast consumption of the mussels may also indicate that zoos may reflect on adding it to the husbandry protocols and diet of these birds, as it can be something valuable to the animals at a nutritional level [60]. On the other hand, among the EE applied, the sensory EE, the vocalizations, needs more caution, since it only aroused a greater degree of attention and resting behaviors, which may indicate either curiosity towards the new sound or an increase in stress. The behavior of this group of ibises changes with time of day, with most of the behaviors decreasing activity towards the end of the day. The increased behaviors towards the end of the day may provide information for future studies, as depending on the observed behaviors, it may indicate that it will still increase the activity later in the afternoon. There are some similarities between the behavior of wild ibises and the one performed by this study’s flock. Together with the absence of abnormal behavior, this suggests the apparent good welfare of the flock, even if physiological parameters are absent. Regarding this species in the wild, this study can shed light on some behaviors that were not yet described, and, to our understanding, it is the first attempt to build an ethogram for this species, which can be used and updated with birds both in captivity and in the wild. This study has an impact on the lives of this group of ibises, as some of the EE types used can be essential for the ecology of this species. The change in the volume of water in the lakes is one example, as it mimics what happens in the wild and can give important social cues regarding the breeding season. If activity levels increase and stimulate behaviors such as exploration, we suggest that zoos integrate this “tidal” change of lakes as much as possible for each facility so the habitat remains relevant for the ecology of these birds.

## Figures and Tables

**Table 1:** Ethogram for the scarlet ibis adapted from Spiezio and colleagues [33]. The behaviors that were observed in this data collection but were not described in the original ethogram are represented in bold. The asterisks represent behaviors classified as events [32].

Type of Behaviour	Behaviour	Behavior’s Description
**Solitary**	Attentive	Being vigilant, scanning the environment and listening or watching something in the surroundings.
	Preening	Using the bill to straighten and clean their feathers; scratching their body (neck and head) using their feet; bathing and sunbathing (fanning the wings to warm up in the sun).
	Flight *	Flying or hopping in the habitat.
	Walking	Walking around the habitat.
	Maintenance	Eating the food provided in the feeding points around the habitat or provided by the caretakers in feeding sessions and drinking.
	Resting	Standing on one or both legs, with the head turned back and tucked beneath the wings.
	Foraging	Looking for and manipulating food by probing the lake, ground, tree branches, crevices, and other elements of the habitat with the bill. (Walking slowly and frequently makes short pecks into the ground).
	**Exploration**	Showing curiosity towards their environment (mostly associated with novelty).
	**Stationary**	Standing motionless on one or both legs with the head down and still
**Social affiliative**	**Begging**	Approaching an adult and rhythmically begs by bobbing the head and touching the adult with the bill (behavior only exhibited by offspring).
	Preening others	Tidying and cleaning the feathers of a conspecific with the bill.
	Other affiliative *	Greeting displays include head tossing, head rubbing, mutual bill shaking; and observing conspecifics.
**Social agonistic**	Aggression *	Pecking towards approaching birds, hitting a conspecific with the bill or with the legs.
	Agonistic display *	Bill gaping, ruffling the feathers, moving or lunging toward conspecifics, touching slightly with the tip of the bill.
	Stealing *	Grabbing a piece of food from another bird and sometimes pecking the bird.
**Acoustic**	Vocalizations *	Emission of vocalizations/sounds by the ibises

**Table 2:** Schedule of the data collection from the baseline condition to the post-enrichment with devices, the last condition. Each day, represented by X, included a total of 3 h through morning, midday, and afternoon sessions.

	*January*						*February*								*March*						
	6	7	8	26	28	30	5	7	9	15	17	19	25	27	1	9	10	11	19	20	21
*Baseline*	X	X	X																		
*Physical enrichment*				X	X	X															
*Nutritional enrichment*							X	X	X												
*Sensorial enrichment*										X	X	X									
*Tactile enrichment*													X	X	X						
*Post enrichment*																X	X	X			
*Post enrichment with devices*																			X	X	X

**Table 3:** Mean times spent on each behavior for each condition. The last three behaviors; vocalizations, flight, and general agonistic display are event behaviors, while the remaining ones are states. W in the table refers to Kendall’s W for effect size.

	Condition	Baseline	Physical Enrichment	Nutritional Enrichment	Sensory Enrichment	Tactile Enrichment	Post-Enrichment	Post-Enrichment with Devices	X^2^ (6, 1116)	*p*-Value	W
Behaviour											
Attentive		0.19	0.10 *	0.46 *	0.79 *	0.21	0.21	0.22	518.185	<0.001	0.077
Preening		1.25 ^abcde^	1.75 ^afgh^	1.23 ^fijkl^	1.41 ^bgimn^	1.38 ^chjop^	1.62 ^dkmo^	1.70 ^elnp^	227.475	<0.001	0.034
Walking		0.25 ^ab^	0.14 ^acd^	0.15 ^bef^	0.10 *	0.22 ^ce^	0.26 ^df^	0.06 *	181.145	<0.001	0.027
Maintenance		0.37	0.18 *	0.34 ^a^	0.30 ^bc^	0.39 ^b^	0.03 *	0.42 ^ac^	264.392	<0.001	0.039
Foraging		0.99 ^abcde^	1.00 ^fgh^	0.66 ^afij^	0.82 ^bikl^	0.67 ^cgkm^	0.84 ^djmn^	0.73 ^ehln^	109.464	<0.001	0.016
Resting		0.18 ^abc^	0.20 ^def^	0.19 ^ghi^	0.27 ^adgj^	0.28 ^behk^	0.27 ^cfil^	0.21 ^jkl^	42.513	<0.001	0.006
Exploration		0.00 ^ab^	0.14 *	0.05 *	0.00 ^cd^	0.22 ^ace^	0.00 ^ef^	0.26 ^bdf^	528.049	<0.001	0.079
Stationary		0.71	0.40 *	0.82	0.27 *	0.58 *	0.67	0.33 *	452.144	<0.001	0.068
Vocalizations		0.00 ^abc^	0.00 ^def^	0.00 ^ghi^	0.00 ^jkl^	0.01 ^adgjm^	0.02 ^behkm^	0.02 ^cfil^	95.300	<0.001	0.014
Flight		0.03 ^a^	0.03 ^bc^	0.04 ^bd^	0.01 ^adefg^	0.04 ^e^	0.05 ^cf^	0.05 ^g^	22.470	<0.001	0.003
General agonistic display		0.02 ^a^	0.01 ^bc^	0.03 ^bd^	0.02 ^e^	0.00 ^adefg^	0.03 ^cf^	0.03 ^g^	28.204	<0.001	0.004

**Note**: The letters “a,” to “p” indicate pairs with significant differences in the pairwise comparisons; significance level *p* = 0.05. The * indicates significant differences between all the pairwise comparisons for that behavior.

## Data Availability

Data will be provided upon request.

## Supplementary Materials

The following supporting information can be downloaded at: https://www.mdpi.com/article/10.3390/ani14131903/s1, Table S1: Species that share the habitat with the group of scarlet ibises; Figure S1: Representation of the high (left), intermediate (center) and low tide (right) in small (top) and big (bottom) lakes in the habitat.
