# Supplementary material for: The Effects of Enrichment on Zoo-Housed Scarlet Ibis Behavior
Source: Animals (Basel). 2024 Jun 27;14(13):1903. doi: 10.3390/ani14131903 (PMC11240375; doi:10.3390/ani14131903)
Supplement: Supplementary file 1 [file animals-14-01903-s001.zip › animals-3023641-supplementary.pdf]

Table S1. Species that share the habitat with the group of scarlet biises.

Species	Common name	Number of individuals
Mareca sibilatrix	Chiloe wigeon	4
Aramides ypecaha	Giant wood rail	2
Athene cunicularia	Burrowing owl	7
Butorides virescens	Green heron	4
Eurypyga helias	Sunbittern	2
Guira guira	Guira cuckoo	25
Meleagris ocellata	Ocellated turkey	1
Nycticorax nycticorax	Black-crowned night-heron	5
Platalea ajaja	Roseate spoonbill	2
Plegadis falcinellus	Glossy ibis	6
Pteroglossus viridis	Green araçari	2
Iguana iguana	Green iguana	1
Trachemys scripta elegans	Red-eared slider	2

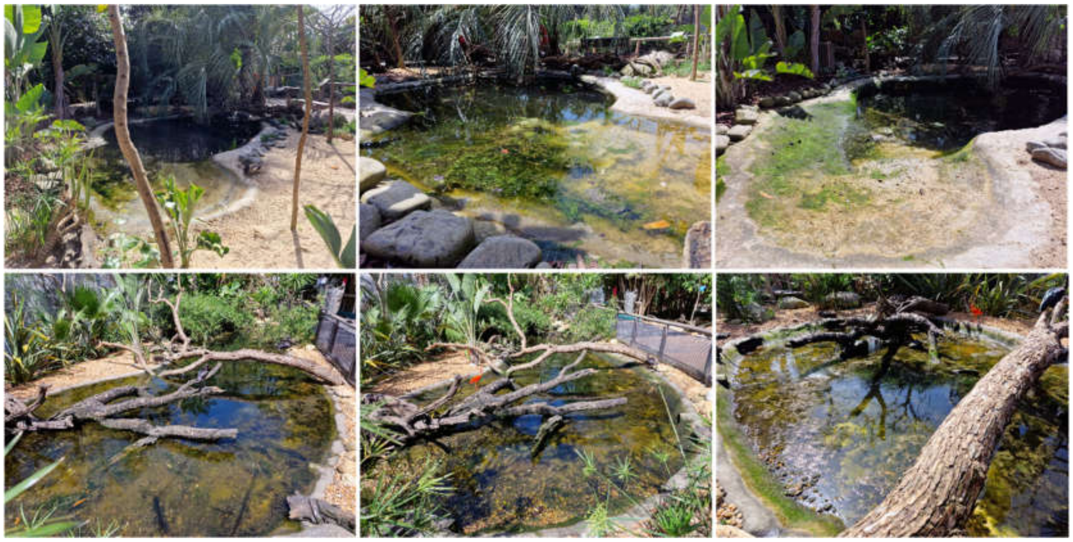

Figure S1. Representation of the high (left), intermediate (center), and low tide (right) in small (top) and big (bottom) lakes in the habitat.
